# Personalized CFTR Modulator Therapy for *G85E* and *N1303K* Homozygous Patients with Cystic Fibrosis

**DOI:** 10.3390/ijms241512365

**Published:** 2023-08-02

**Authors:** Simon Y. Graeber, Anita Balázs, Niklas Ziegahn, Tihomir Rubil, Constanze Vitzthum, Linus Piehler, Marika Drescher, Kathrin Seidel, Alexander Rohrbach, Jobst Röhmel, Stephanie Thee, Julia Duerr, Marcus A. Mall, Mirjam Stahl

**Affiliations:** 1 Department of Pediatric Respiratory Medicine, Immunology and Critical Care Medicine and Cystic Fibrosis Center, Charité–Universitätsmedizin Berlin, Corporate Member of Freie Universität Berlin and Humboldt-Universität zu Berlin, 13353 Berlin, Germany; 2 German Centre for Lung Research (DZL), Associated Partner Site, 13353 Berlin, Germany; 3 Berlin Institute of Health (BIH) at Charité–Universitätsmedizin Berlin, 10117 Berlin, Germany

**Keywords:** cystic fibrosis, CFTR, CFTR modulator, G85E, N1303K, human nasal epithelial cells, intestinal current measurement, nasal potential difference

## Abstract

CFTR modulator therapy with elexacaftor/tezacaftor/ivacaftor (ETI) has been approved for people with CF and at least one F508del allele in Europe. In the US, the ETI label has been expanded to 177 rare *CFTR* mutations responsive in Fischer rat thyroid cells, including *G85E*, but not *N1303K*. However, knowledge on the effect of ETI on G85E or N1303K CFTR function remains limited. In vitro effects of ETI were measured in primary human nasal epithelial cultures (pHNECs) of a *G85E* homozygous patient and an *N1303K* homozygous patient. Effects of ETI therapy in vivo in these patients were assessed using clinical outcomes, including multiple breath washout and lung MRI, and the CFTR biomarkers sweat chloride concentration (SCC), nasal potential difference (NPD) and intestinal current measurement (ICM), before and after initiation of ETI. ETI increased CFTR-mediated chloride transport in *G85E*/*G85E* and *N1303K*/*N1303K* pHNECs. In the *G85E*/*G85E* and the *N1303K*/*N1303K* patient, we observed an improvement in lung function, SCC, and CFTR function in the respiratory and rectal epithelium after initiation of ETI. The approach of combining preclinical in vitro testing with subsequent in vivo verification can facilitate access to CFTR modulator therapy and enhance precision medicine for patients carrying rare *CFTR* mutations.

## 1. Introduction

Cystic fibrosis (CF) is an autosomal recessive disease caused by mutations in the cystic fibrosis transmembrane conductance regulator (*CFTR*) gene [1]. As a consequence, the CFTR protein, which acts as an ion channel in various epithelia in the human body, is substantially reduced in quantity and/or quality, leading to an impaired function of the chloride channel [2]. This results in thickened secretions of multiple organs, including the airways, ultimately leading to damage of the affected organs and loss of their function. For a long time, therapeutic strategies aimed to reduce symptoms of organ dysfunction. The emergence of highly effective CFTR modulator therapies has transformed the clinical landscape of CF care. Novel triple combination elexacaftor/tezacaftor/ivacaftor (ETI) therapy is now available for ~90% of people with CF carrying at least one allele of the most common mutation *F508del* [3,4,5,6,7,8,9]. ETI therapy leads to substantial clinical benefits and improvement in CFTR function to 40 to 50% of normal CFTR activity measured by nasal potential difference (NPD), intestinal current measurement (ICM), lung clearance index (LCI) and lung morphology in real world observational trials in patients with CF and at least one *F508del* mutation [10,11]. Additionally, the FDA approved ETI for 177 rare *CFTR* mutations, based on in vitro data in Fischer rat thyroid cells. However, there is still an unmet need to address ~10% of people with non-approved mutations or ultra-rare mutations with unknown consequences, where personalized medicine approaches could become useful to enhance their accessibility to approved CFTR modulator therapies [12,13]. Here, we report an approach for individualized treatment for two patients with CF, homozygous for the *G85E* and *N1303K* mutation and for whom CFTR modulator therapy is not available in Europe, as both conditions are not approved for ETI by the European Medicines Agency (EMA) (although *G85E* has been approved by the FDA).

Both *N1303K* and *G85E* are classified as processing mutations, which are refractory to lumacaftor [14,15]; with *N1303K* additionally being classified as a gating mutation [15,16]. However, recent reports from investigations of airway epithelial cells suggest that G85E and N1303K CFTR proteins are responsive to ETI [17,18]. Moreover, CF patients homozygous for rare *CFTR* mutations provide an opportunity to study the effect of specific mutations on CFTR function in a translational disease model system, which is not possible in compound heterozygous patients with CF. Furthermore, limited clinical data are available on the in vivo benefits of ETI for patients carrying these mutations [13,19,20,21].

In this study, we first utilized patient-derived primary human nasal epithelial cultures (pHNECs) from one *G85E* homozygous patient and one *N1303K* homozygous patient as a tool to predict the clinical response to ETI therapy as a precision medicine model in vitro. In addition, we investigated the effect of ETI therapy in these patients on in vivo CFTR function with the CFTR biomarkers sweat chloride concentration (SCC), NPD, and ICM as well as clinical outcomes, including FEV_1_ % predicted and sensitive endpoints for lung structure and function, such as LCI and magnetic resonance imaging (MRI) of the lung.

## 2. Results

### 2.1. Case Presentations

The *G85E*/*G85E* patient is a 15-year-old female diagnosed with CF at the age of two years secondary to recurrent respiratory infections and a failure to thrive. SCC at that time was clearly pathologic with 77 mmol/L and a genetic analysis that demonstrated homozygosity of *G85E* in the *CFTR* gene. Symptomatic therapies were initiated according to the European Cystic Fibrosis Society guidelines, including bronchodilator and mucolytics inhalation with airway clearance, oral pancreatic enzyme replacement therapy, and fat-soluble vitamin supplementation. This led to an improvement in thriving, but the girl soon experienced a first and then recurrent episodes of allergic bronchopulmonary aspergillosis (ABPA). Therefore, she required extended symptomatic therapy including recurrent cycles of systemic and inhaled glucocorticosteroids during childhood and adolescence. Recurring ABPA episodes were treated with anti-IgE antibodies without clear response in clinical parameters. Under this treatment, the patient suffered mild-to-moderate obstructive lung disease at age 14 years and had already shown advanced structural CF lung disease in chest MRI (Table 1). Continuous systemic antifungal therapy led to a deceleration of lung function decline; however, the patient still reported daily cough with both phases of persistent dry cough and extensive sputum production. In addition, bronchial tightness necessitated the frequent use of bronchodilators. In January 2021, our patient experienced a new significant decline in lung function during a pulmonary exacerbation that was only partly restored following intravenous antibiotic treatment.

The *N1303K*/*N1303K* patient is a 19-year-old male who was also diagnosed at preschool age due to recurrent pulmonary infections and failure to thrive. Issues with adherence to symptomatic therapy during adolescence added to the clear progression over time, especially of CF lung disease, with ultimately polymicrobial infection with Methicillin resistant Staphylococcus aureus (MRSA), Pseudomonas aeruginosa and Mycobacterium abscessus, among other pathogens. Repeated Pseudomonas-effective antibiotic therapy cycles did not lead to stabilization of lung disease (Table 1). Besides the impaired pulmonary function, the *N1303K*/*N1303K* patient also suffered from an advanced CF liver disease with pronounced structural changes, but preserved function. However, infection with nontuberculous mycobacteria (NTM) could pose a problem if liver transplantation is required in the future. Therefore, we decided to start an antimycobacterial therapy in April 2021, taking into account the other pathogens present. The initial five-drug systemic NTM therapy with tigecycline, amikacin, cefoxitin, meropenem and clofazimine, in addition to colistin p.i., had to be adjusted in the first months due to limited tolerability, but was then carried out as a quadruple therapy over 9 months, which led to stabilization of the patient. However, with side effects increasing over time and a recurrence of pulmonary exacerbation under therapy, we evaluated the treatment with ETI for this patient.

### 2.2. Preclinical Testing of ETI Therapy In Vitro

**Figure 1:**
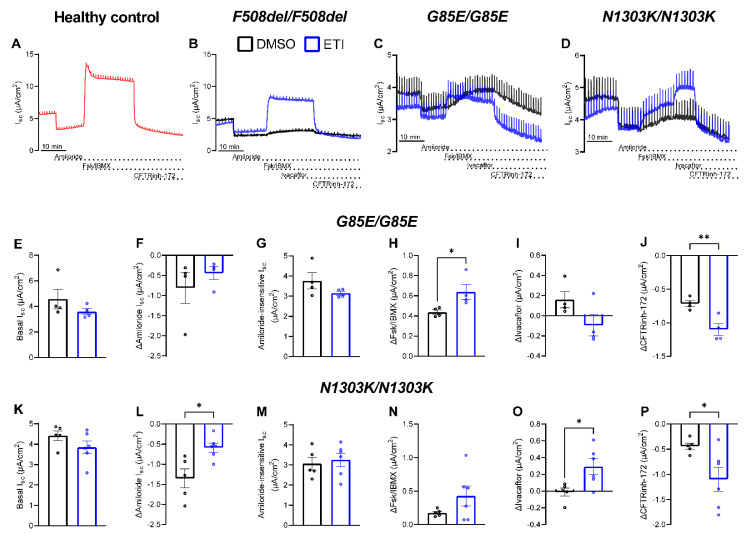
Treatment with elexacaftor/tezacaftor/ivacaftor (ETI) rescues cystic fibrosis transmembrane conductance regulator (CFTR) function in nasal epithelial cultures from the *G85E*/*G85E* and the *N1303K*/*N1303K* patient. (**A**–**D**) Representative tracings of short-circuit transepithelial current (I_SC_) measurements in cultures from a healthy donor (**A**), a CF patient homozygous for *F508del* (**B**), the CF patient homozygous for *G85E* (**C**) and the CF patient homozygous for *N1303K* (**D**). CF cultures were incubated with elexacaftor/tezacaftor or dimethyl sulfoxide (DMSO) for 24 h. (**E**–**J**) Quantification of the basal I_SC_ (**E**), amiloride-sensitive I_SC_ (∆Amiloride) (**F**), amiloride-insensitive I_SC_ (**G**), and effects of cAMP activation (∆Forskolin/IBMX) (**H**), ivacaftor (∆Ivacaftor) (**I**) and CFTR inhibitor-172 (∆CFTRinh-172) (**J**) on I_SC_ in the *G85E*/*G85E* patient. Quantification of the basal I_SC_ (**K**), amiloride-sensitive I_SC_ (∆Amiloride) (**L**), amiloride-insensitive I_SC_ (**M**), and effects of cAMP activation (∆Forskolin/IBMX) (**N**), ivacaftor (∆Ivacaftor) (**O**) and CFTR inhibitor-172 (∆CFTRinh-172) (**P**) on I_SC_ in the *N1303K*/*N1303K* patient. n = 4–6 filters per individual per group, data are presented as mean ± SD. The black tracings and bars represent the vehicle control treated with DMSO and the blue tracings and bars represent the cultures treated with ETI. * *p* < 0.05, ** *p* < 0.01 ETI versus DMSO.

To test the potential benefit of ETI therapy in vitro, we generated highly differentiated airway epithelial cultures from nasal brushings of both patients and performed transepithelial short-circuit current measurements in Ussing chambers (Figure 1). The basal short circuit current (I_sc_), amiloride-sensitive I_sc_ and amiloride-insensitive I_sc_ were not significantly different between vehicle control and ETI treatment in cultures derived from the *G85E*/*G85E* patient, although amiloride-sensitive I_sc_ showed a trend towards a decrease with ETI treatment (Figure 1C,E–G). ETI treatment significantly enhanced CFTR-mediated chloride currents compared with vehicle-treated control, as indicated by forskolin peak response (*p* < 0.05) and CFTRinhibitor-172 inhibited current (*p* < 0.01) in *G85E*/*G85E* pHNECs (Figure 1H,J). Compared with our previously published data from pHNECs from healthy people (mean CFTRinhibitor-172 sensitive current −10.1 µA/mm^2^, n = 31) [22], rescue of CFTR function by ETI treatment, as determined from CFTRinhibitor-172 sensitive current (−1.1 µA/mm^2^), was 11.0% of the normal CFTR activity for pHNECs of the *G85E*/*G85E* patient. In pHNECs derived from the *N1303K*/*N1303K* patient, basal I_sc_ and amiloride-insensitive I_sc_ were unchanged, whereas amiloride-sensitive I_sc_ was significantly reduced by ETI treatment compared with dimethyl sulfoxide (DMSO) control (*p* < 0.05), suggesting decreased activity of the epithelial sodium channel (ENaC) (Figure 1D,K–M). Forskolin-stimulated I_sc_ showed a trend towards an increase upon ETI treatment and acute ivacaftor response was significantly increased with ETI treatment compared with vehicle control (Figure 1D,N,O). Furthermore, ETI treatment significantly increased CFTRinhibitor-172 sensitive current (−1.1 µA/mm^2^) compared with DMSO control (−0.44 µA/mm^2^) in pHNECs from the *N1303K*/*N1303K* patient (*p* < 0.05) corresponding to 11.0% of the normal CFTR activity (Figure 1D,P).

### 2.3. Improvements in CFTR Function In Vivo

**Figure 2:**
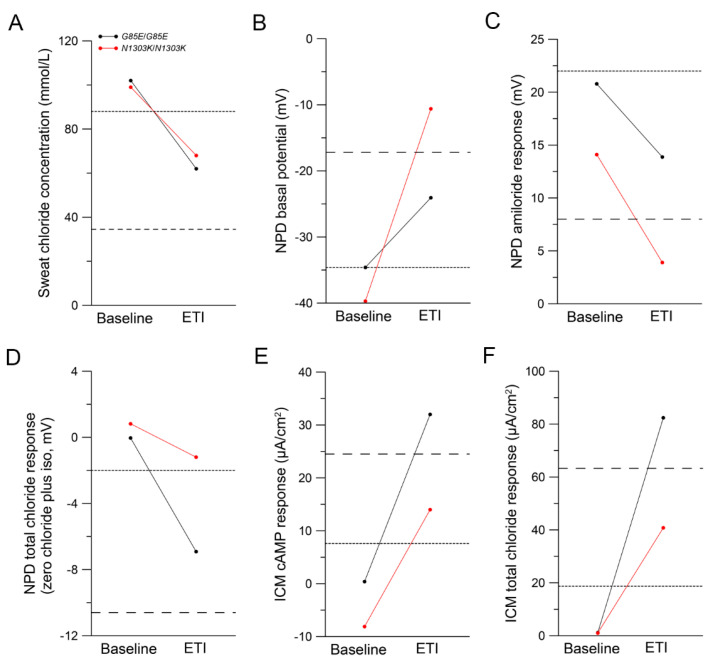
Elexacaftor/tezacaftor/ivacaftor (ETI) therapy improves mutant cystic fibrosis transmembrane conductance regulator (CFTR) function in both the *G85E*/*G85E* and the *N1303K*/*N1303K* patient. (**A**–**F**) Paired measurements of the CFTR biomarkers sweat chloride concentration (**A**), nasal potential difference (NPD) (**B**–**D**), and intestinal current measurement (ICM) (**E**–**F**) in a *G85E*/*G85E* (black) and an *N1303K*/*N1303K* (red) patient at baseline and 3 months (*G85E*/*G85E*) or 2 weeks (*N1303K*/*N1303K*) after initiation of ETI therapy. (**A**) Sweat chloride concentration. (**B**–**D**) NPD basal potential (**B**), NPD amiloride response (**C**), and NPD total chloride response (zero chloride plus isoproterenol (iso) solutions). (**E**,**F**) ICM cAMP response (**E**) and ICM total chloride response obtained by cAMP-dependent stimulation and cholinergic (calcium-dependent) co-activation (**F**). ICM studies were performed in the presence of amiloride and indomethacin. The dotted line represents median values for *F508del/F508del* patients treated with tezacaftor/ivacaftor (n = 41) and the dashed line represents median values for *F508del/F508del* patients treated with elexacaftor/tezacaftor/ivacaftor (n = 44) [11].

Based on the in vitro evidence, ETI was started as an off-label therapy in both patients. To evaluate the in vivo effect of ETI therapy, we measured CFTR function in three different tissues using the established CFTR biomarkers SCC, NPD and ICM at baseline and after initiation of ETI therapy (Figure 2) [11,23,24].

Both patients had a pathological SCC above 60 mmol/L and showed no CFTR function in the respiratory or rectal epithelium at baseline (Figure 2). In the *G85E*/*G85E* patient, SCC was reduced by 40 mmol/L after initiation of ETI therapy (Figure 2A). In the NPD, the basal potential was improved from –34.6 mV at baseline to –24.1 mV after initiation of ETI and the amiloride response improved from 20.8 mV to 13.9 mV (Figure 2B,C). Furthermore, NPD total chloride response improved from 0.0 mV to −6.9 mV corresponding to 32.4% of CFTR activity in the nasal epithelium of healthy people (Figure 2D). In the ICM, we observed a cAMP-dependent chloride secretory response of 32.0 µA/cm^2^ after initiation of ETI therapy in the *G85E*/*G85E* patient, corresponding to rescue of CFTR function in the rectal mucosa to a level of 37.6% of normal (Figure 2E). The total chloride secretory response increased from 1.1 µA/cm^2^ at baseline to 82.4 µA/cm^2^ after initiation of ETI, corresponding to a level of 29.4% of normal CFTR activity (Figure 2F). In the *N1303K*/*N1303K* patient, SCC was reduced by 31 mmol/L after initiation of ETI therapy (Figure 2A). In the NPD, the basal potential was improved from −39.7 mV to –10.6 mV and the amiloride response from 14.1 mV to 3.9 mV (Figure 2B,C). NPD total chloride response improved from 0.8 mV to −1.2 mV, corresponding to 5.5% of normal CFTR activity in the nasal epithelium (Figure 2D). In the ICM, we observed a cAMP-dependent chloride secretory response of 14.0 µA/cm^2^ after initiation of ETI therapy in the *N1303K*/*N1303K* patient, corresponding to rescue of CFTR function in the rectal mucosa to a level of 16.5% of normal (Figure 2E). The total chloride secretory response increased from 1.0 µA/cm^2^ to 40.8 µA/cm^2^, corresponding to a level of 14.6% of normal CFTR activity (Figure 2F).

### 2.4. Clinical Improvements following Treatment with ETI

**Figure 3:**
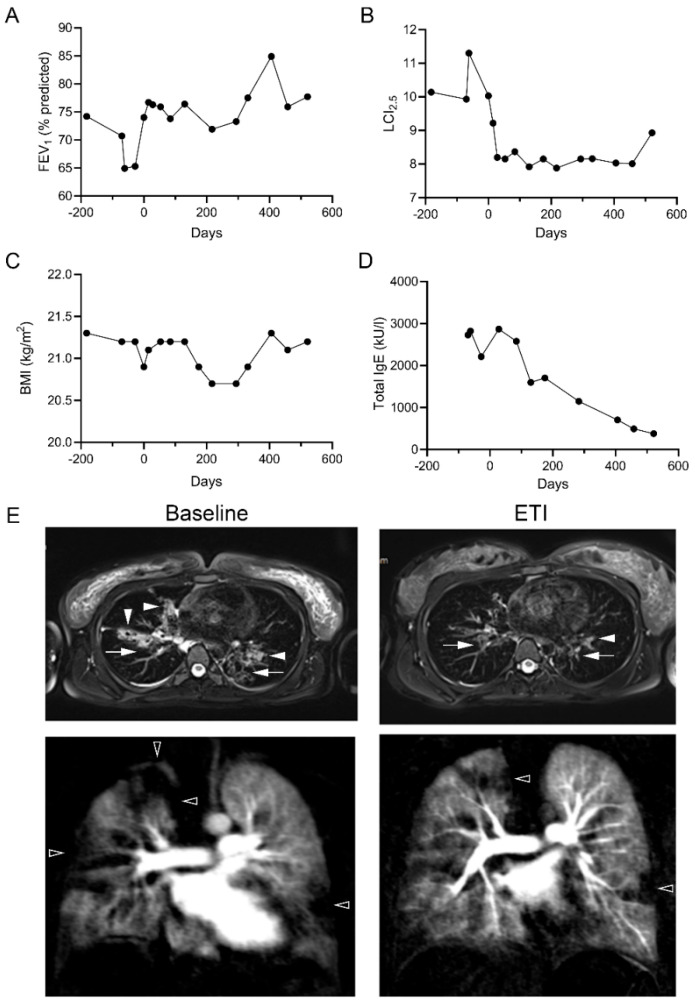
Elexacaftor/tezacaftor/ivacaftor (ETI) therapy improves clinical outcomes in the *G85E*/*G85E* patient. (**A**–**D**) Repeated measurements of FEV1% predicted (**A**), lung clearance index (LCI_2.5_) (**B**), BMI (**C**) and total immunoglobulin E (IgE) from 200 days before and 600 days after initiation of ETI. (**E**) Representative MRI image at baseline and after initiation of ETI. Structural airway abnormalities (wall thickening and/or bronchiectasis) are indicated by arrows and mucus plugging by white arrowheads. Perfusion abnormalities are indicated by black arrowheads.

**Figure 4:**
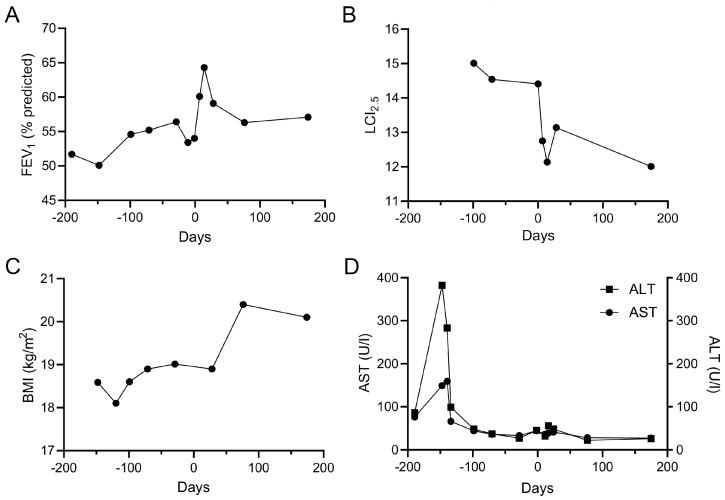
Elexacaftor/tezacaftor/ivacaftor (ETI) therapy improves clinical outcomes in the *N1303K*/*N1303K* patient. (**A**–**D**) Repeated measurements of FEV1% predicted (**A**), lung clearance index (LCI_2.5_) (**B**), BMI (**C**), aspartate transaminase (AST, GOT) and alanine transaminase (ALT, GPT) (**D**) from 200 days before and 200 days after initiation of ETI.

The initiation of therapy with ETI resulted in prompt stabilization of the clinical status of both patients, with a particular improvement in FEV_1_ % predicted and LCI (Figure 3A,B and Figure 4A,B). The *G85E*/*G85E* patient stayed in the normal range for BMI after initiation of ETI (Figure 3C). However, this patient showed a reduction in total serum IgE despite stopping all ABPA-related therapies (Figure 3D). Furthermore, we observed substantial improvements in mucus plugging and bronchial wall thickening in MRI images of the lung after initiation of ETI (Figure 3E).

In the *N1303K*/*N1303K* patient, we were able to stop the NTM therapy after start of ETI without a decline in respiratory status (Figure 4A,B). Furthermore, an improvement and stabilization in weight into the normal BMI range appeared that was not achieved in previous years (Figure 1C). For safety monitoring, our patients received ophthalmology exams and routine hepatic function monitoring. In this context, transaminase levels showed improvements after initiation of ETI. Until now, neither the *G85E*/*G85E* patient who had been treated with ETI for 50 months, nor the *N1303K*/*N1303K* patient who had been treated for 16 months have shown signs of side effects and have reported no other adverse reactions after initiation of ETI.

## 3. Discussion

This study provides a comprehensive assessment of the effects of ETI on CFTR function in pHNECs of a *G85E* homozygous patient and an *N1303K* homozygous patient in vitro as well as of the improvement of CFTR function in vivo after initiation of ETI in both patients. We were able to show that ETI increased CFTR-mediated currents in pHNECs of both patients (Figure 1). As predicted by the preclinical results, the in vivo CFTR function of the *G85E*/*G85E* patient improved to 30–40% of normal CFTR activity in the respiratory and rectal epithelium after initiation of ETI. The *N1303K*/*N1303K* patient showed an improvement to 5–20% in the respiratory and rectal epithelium after initiation of ETI (Figure 2). In both patients, partial restoration of CFTR function led to improvements in clinical outcomes after initiation of ETI (Figure 3 and Figure 4).

The *G85E* mutation is classified as a class II missense mutation, resulting in decreased quantity and function of CFTR protein. In the US, therapy with ETI is approved for patients with CF carrying the *G85E* mutation based on in vitro data in Fischer rat thyroid cells. In Europe, ETI is not approved for patients carrying the *G85E* mutation (without *F508del* on the other allele) due to the lack of clinical studies investigating efficacy and safety of this therapy in vivo. It has been previously shown that ETI improves CFTR function in rectal organoids of a *G85E* heterozygous patient with a stop codon on the other allele [25]. We observed a consistent rescue of CFTR function by ETI in the pHNEC of the *G85E*/*G85E* patient. Of note, acute addition of ivacaftor did not further potentiate cAMP-stimulated chloride secretion in the *G85E*/*G85E* cultures and even tended to reduce the transepithelial current, indicating that the potentiator ivacaftor may not be essential for optimizing the chloride channel function of this specific trafficking mutation. However, to confirm this observation, the acute and chronic effects of ivacaftor on the G85E mutation need to be assessed in a larger sample size in future studies. Furthermore, by additionally measuring in vivo CFTR biomarkers, our study shows that ETI improves CFTR function in the *G85E* homozygous patient to levels comparable to ETI in *F508del* homozygous patients [11]. Overall, these data suggest that ETI is beneficial in patients with a *G85E* mutation.

*N1303K*, the fourth most common *CFTR* mutation, is also classified as a class II missense mutation. Due to a lack of improvement above a threshold of 10% WT CFTR function after treatment with ETI in Fischer rat thyroid cells [26,27], ETI is not approved for treatment in patients carrying the *N1303K* mutation (without another responsive mutation on the other allele) in any country worldwide. Recent evidence suggests that, in addition to impaired CFTR trafficking, the *N1303K* mutation also causes a severe gating defect in CFTR [16]. Consistent with these data, our results in pHNEC suggest that potentiation by ivacaftor is an important mechanism in rescuing N1303K–CFTR function. This observation is supported by a study in *N1303K*/*N1303K* rectal organoids showing that potentiation by ivacaftor is important for reaching therapeutically relevant levels of organoid swelling after pre-treatment with elexacaftor and tezacaftor [25]. Recently, it has been suggested that patients with an *N1303K* mutation could clinically benefit from ETI therapy [13,19,21]. This is further supported by our study showing improvement of CFTR function in the *N1303K*/*N1303K* patient in the range of tezacaftor/ivacaftor in *F508del* homozygous patients [11]. However, in previous studies, *N1303K* heterozygous and homozygous patients treated with ETI showed substantial improvements in lung function despite only a modest decrease in SCC [13,19,21]. The large decrease in SCC in the *N1303K*/*N1303K* patient in our study may partially be explained by the homozygous genotype. However, larger studies with *N1303K* homozygous and heterozygous patients are needed to address the question of a gene dosage effect for the *N1303K* mutation. Nevertheless, the relatively large improvement in lung function compared with the relatively modest improvement in sweat chloride in previous studies may suggest tissue-specific expression and therapeutic rescue of N1303K, indicating that SCC might not be the ideal biomarker to assess the response to therapy on the functional level therapy in patients carrying the *N1303K* mutation. This question needs to be addressed in future clinical trials and the in vivo CFTR biomarker NPD and ICM may help to solve this conundrum regarding N1303K. Interestingly, we further observed reduced amiloride-sensitive currents in ETI treated pHNECs of the *N1303K*/*N1303K* patient, suggesting decreased activity of ENaC. This observation may be explained by the restoration of a previously described functional interaction between CFTR and ENaC at the plasma membrane, leading to downregulation of ENaC-mediated sodium/fluid absorption and thus improved airway surface hydration and mucociliary clearance [28,29,30,31]. This is consistent with observations in the NPD of this patient showing a substantially lower basal transepithelial potential difference and a reduced amiloride response on ETI. In previous studies, improvements in basal potential difference and amiloride response were only observed for ETI in patients with at least one *F508del* allele, but not for tezacaftor/ivacaftor or lumacaftor/ivacaftor in *F508del* homozygous patients [11,23]. In pHNECs, we observed residual currents after inhibition of ENaC and CFTR at the end of the experiment. These residual currents were previously observed in freshly excised native nasal epithelium [32,33] and may be explained by (i) non-CFTR mediated anion conductance, such as SLC26A9 or TMEM16A; (ii) amiloride-insensitive sodium conductance; or (iii) passive chloride movement due to the chloride gradient applied under these experimental conditions (145 mM basolateral to 5 mM apical) [34]. Taken together, these data suggest that different preclinical models should be investigated, the cut-off in Fischer rat thyroid cells may need to be adjusted and the combination with in vivo testing may help to establish in vivo efficacy in patients with uncommon *CFTR* mutations.

In pHNECs from the *G85E*/*G85E* and the *N1303K*/*N1303K* patient, we observed an improvement of in vitro CFTR function to about 10% of CFTR activity of healthy controls. However, in vivo CFTR function was improved to 30–40% of normal CFTR activity in the *G85E*/*G85E* patient and to 5–20% in the *N1303K*/*N1303K* patient. This finding shows that patient-derived models such as pHNECs enable detection of functional restoration of CFTR variants in vitro but cannot predict the degree of the in vivo response in an individual patient. This has also previously been observed for other preclinical models, such as intestinal organoids, where no correlation between in vitro organoid swelling and in vivo improvement in CFTR function was observed in F508del homozygous patients treated with lumacaftor/ivacaftor [35]. We therefore suggest that in vivo biomarkers provide a valuable tool to confirm findings in preclinical in vitro models in individual patients.

Our study has several limitations: First, the small sample size may limit generalizability of the data. In n-of-1 studies, the variability of clinical endpoints could affect or disguise potential therapeutic effects. The intraindividual variability of repeated short-term measurements has been previously reported at 6.3% for FEV_1_ % predicted and 7.4% for LCI [36,37]. The *G85E*/*G85E* patient showed an improvement in lung function in repeated measurements after initiation of ETI with a mean improvement of +6.6% in FEV_1_ % predicted and −2.1 (20.2%) in LCI when comparing the mean values before and after initiation of ETI. In the *N1303K*/*N1303K* patient, FEV_1_ % predicted improved initially by +10.3% and LCI improved by −2.3 (15.7%). Although FEV_1_ % predicted decreased and LCI increased again, both parameters were still improved compared with the repeated measurements before initiation of ETI. Previous studies in *N1303K* patients have reported higher increases in FEV_1_ % predicted after initiation of ETI treatment than observed in our study [13,19,21]. Clinical trials and real-world studies assessing the effects of ETI in patients with at least one F508del mutation have also observed a high heterogeneity in the response in FEV_1_ % predicted, which may be explained by the fact that FEV_1_ is influenced by numerous factors independent of CFTR function, such as fixed airflow limitation due to irreversible structural lung damage or adherence to concomitant therapies. We used multiple repeated clinical outcome measures and independent measurements of the in vitro and in vivo CFTR function to determine the response to ETI in both patients, suggesting that the improvement in lung function is related to the therapy rather than variability of the measurements. In addition, other recent reports support that ETI shows clinical benefit in patients with a *G85E* and an *N1303K* mutation [13,19,21,38]. Second, pHNECs have not been validated in large patient cohorts receiving CFTR modulator therapy and it is unknown if a lack of response in pHNECs predicts a lack of clinical response. Third, for the in vitro experiments, we used a concentration of tezacaftor that was previously used in the pre-clinical development of the triple combination therapy [39], although it is unknown if these concentrations are reached in patients. Finally, the elexacaftor used in the in vitro experiments is a racemic mixture of two enantiomers which may have different effects than those of ETI in patients and therefore potentially underestimate the in vitro effects [18,40].

In summary, our results show an improvement of CFTR function and clinical benefit in patients homozygous for *G85E* or *N1303K* after initiation of ETI therapy. Furthermore, these results support that patient-derived pHNECs may have the potential to indicate clinical response of rare *CFTR* mutations to ETI therapy. We suggest that the sequential use of in vitro testing of CFTR function in pHNECs and subsequent in vivo testing using biomarkers of CFTR function, in combination with clinical outcome measures, is a promising approach to facilitate access to CFTR modulator therapy and to enhance precision medicine for patients with CF carrying rare *CFTR* mutations.

## 4. Materials and Methods

### 4.1. Study Design and Participants

Patients participated in an ongoing multicenter longitudinal study on the effects of CFTR modulators (ClinicalTrials.gov Identifier: NCT04732910). The study was approved by the ethics committee of the Charité-Universitätsmedizin Berlin (EA2/220/18). Written informed consent was obtained from both patients included in the study and the parents of the *G85E/G85E* patient. Patients provided nasal brushes for preclinical testing in primary nasal epithelial cell cultures. After proof of a significant preclinical response, the patients were treated with the standard dose of ELX 200 mg and TEZ 100 mg every 24 h in combination with IVA 150 mg every 12 h. Anthropometrics, spirometry, multiple-breath washout, SCC, NPD and ICM were assessed at baseline and after initiation of ETI. In the *G85E/G85E* patient, total IgE levels and lung MRI were additionally performed at baseline and after initiation of ETI.

### 4.2. Nasal Epithelial Cell Cultures

Primary human nasal epithelial cells were cultured as previously described [22,41]. Briefly, cells were obtained by nasal brushing and expanded in co-culture with irradiated mouse 3T3 fibroblasts in the presence of RhoA kinase inhibitor Y-27632 (Stemcell Technologies, Cologne, Germany). Epithelial cells were seeded at passage 2 at a density of 200,000 cells/cm^2^ on human placental type IV collagen-coated Snapwell supports (Corning, Glendale, NY, USA) in UNC–ALI medium and differentiated at the air–liquid interface (ALI) for at least 3 weeks.

### 4.3. Ussing Chamber Experiments in pHNECs

Cultures were treated with 3 µM elexacaftor (E) and 18 µM tezacaftor (T) or with 0.07% dimethyl sulfoxide (DMSO) (vehicle control) for 24 before Ussing chamber analysis. Ivacaftor was added acutely during transepithelial short-circuit current (I_sc_) measurements to ET pre-treated cultures, to provide conditions that maximally enhance CFTR function. CFTR modulators were obtained from Selleck Chemicals (Planegg, Germany), DMSO was obtained from Sigma-Aldrich (St Louis, MO, USA). I_sc_ was measured by EasyMount Ussing chambers (Physiologic Instruments, San Diego, CA, USA) as previously described, in presence of a chloride gradient (basolateral 145 mM vs. apical 5 mM) [22,42]. After 10–20 min equilibration, amiloride (100 µM) was added to inhibit sodium absorption via the epithelial sodium channel (ENaC). To assess CFTR-mediated chloride secretion, forskolin (Fsk, 10 µM) and 3-isobutyl-1-methylxanthin (IBMX, 100 µM) were applied together, then ivacaftor (2.5 µM) was added, followed by CFTR inhibitor-172 (CFTRinh-172, 20 µM, TargetMol chemicals, Boston, MA, USA). All Ussing chamber chemicals and reagents, apart from CFTRinh-172 and ivacaftor, were obtained from Sigma-Aldrich (St Louis, MO, USA) at the highest level of available purity. Bioelectric responses were quantified by LabChart8 (AF Instruments, Oxfordshire, UK).

### 4.4. Lung Function and Multiple-Breath Washout (MBW) Measurements

Spirometry was performed according to the standards endorsed by the American Thoracic Society and European Respiratory Society and values were expressed as percent predicted according to published normative data [43,44]. MBW testing was performed with the Exhalyzer D system (Eco Medics, Duernten, Switzerland) and 100% oxygen was used to wash out resident nitrogen from the lungs with a mouthpiece as interface [45,46]. All measurements were evaluated using spiroware 3.3.1 (Eco Medics, Duernten, Switzerland) [10,47].

### 4.5. Morpho-Functional Chest Magnetic Resonance Imaging (MRI)

A standardized MRI protocol was used on a 1.5 Tesla MRI scanner (Magnetom Avanto, Magnetom Aera; Siemens Healthineers, Erlangen, Germany) at baseline and after initiation of therapy to acquire T1- and T2-weighted sequences. Lung perfusion was assessed by contrast-enhanced imaging [48]. Images were assessed for abnormalities in lung morphology and perfusion using a dedicated morpho-functional MRI score as previously described [49]. In this MRI score, structural findings are assessed by the MRI morphology score that comprises subscores for (i) bronchial wall abnormalities (wall thickening and/or bronchiectasis), (ii) mucus plugging, (iii) abscesses and/or sacculations, (iv) consolidations, and (v) special findings such as pleural effusion. The extent of these structural findings, as well as of changes in lung perfusion, are rated in each lobe (right upper lobe, right middle lobe, right lower lobe, left upper lobe, lingula, left lower lobe) as 0 (no abnormality), 1 (<50% of the lobe involved), or 2 (≥50% of the lobe involved) resulting in a range of the MRI global score from 0 (normal) to 72 (severely abnormal) with each score of ≥1 being rated as abnormal [48,49].

### 4.6. Sweat Chloride

Sweat tests were performed according to the German national diagnostic guideline [50] and the guidelines of the Clinical and Laboratory Standards Institute [51]. Sweating was stimulated by pilocarpine iontophoresis and samples were collected with the Macroduct^®^ system (Model 3700, Wescor, Logan, UT, USA). SCC was measured in a minimum volume of 30 μL using a chloridometer (KWM 20 Chloridometer, Kreienbaum, Langenfeld, Germany).

### 4.7. NPD Measurements

NPD measurements were performed as previously described [23,52,53]. The percentage of normal CFTR activity in the nasal epithelium was defined as the ratio of the total chloride conductance (TCC) of the individual CF patient to the median TCC in age-matched non-CF controls as previously described [23].

### 4.8. Intestinal Current Measurements

ICM was performed as previously described [11,23,24,54]. In brief, biopsies of the rectal mucosa were collected by endoscopic forceps biopsy, immediately stored in tissue medium (medium 199 containing Hank’s salts, L-glutamine and 25 mM HEPES complemented with 5 mM glycine and 0.5 mM sodium-DL-β-hydroxybutyrate or RPMI-1640 medium with L-glutamine and sodium bicarbonate) and mounted in custom-made perfused micro-Ussing chambers. The luminal and basolateral compartments were perfused continuously with a buffer solution of the following composition: 145 mM NaCl, 0.4 mM KH_2_PO_4_, 1.6 mM K_2_HPO_4_, 5 mM D-glucose, 1 mM MgCl_2_, 1.3 mM Ca-gluconate, pH 7.4, at 37 °C. All reagents were obtained from Sigma-Aldrich at the highest level of available purity. Experiments were performed under open circuit conditions. Transepithelial voltage (V_te_) was recorded and transepithelial resistance (R_te_) was determined by applying intermittent (1 s) current pulses (ΔI = 0.5 µA). The equivalent short-circuit current (I_sc_) was determined from continuous V_te_ and R_te_ recordings according to Ohm’s law (I_sc_ = V_te_/R_te_) after appropriate correction for fluid resistance [34,55,56,57]. The percentage of normal CFTR function for each CF patient was calculated by dividing the individual cAMP-induced Isc of the patient by the median cAMP-induced Isc of age-matched non-CF controls as previously described [11,23,24].

### 4.9. Statistical Analysis

Data were analyzed using GraphPad Prism version 9.4.1 (GraphPad Software, San Diego, CA, USA) and R 3.6.2 [58]. Data were normally distributed and are presented as mean and standard deviation. Differences between the ETI and vehicle group were tested by Student’s *t*-test, *p* < 0.05 was accepted to indicate statistical significance.

## Figures and Tables

**Table 1:** Clinical characetristics of the two patients at baseline.

Clinical Characteristic	*G85E*/*G85E*	*N1303K*/*N1303K*
Age (years)	15.0	19.8
Sex	Female	Male
Pancreatic insufficiency	Yes	Yes
Sweat chloride (mmol/L)	102.0	99.0
FEV_1_ (% predicted)	74.0	54.0
LCI_2.5_	10.0	14.5
BMI (kg/m^2^)	20.9	19.0

Definition of abbreviations: FEV_1_ = forced expiratory volume in one second; LCI_2.5_ = lung clearance index; BMI = body mass index.

## Data Availability

The data that support the findings of this study are available on request from the corresponding author M.S. The data are not publicly available due to legal restrictions, e.g., they contain information that could compromise the privacy of research participants.
